# Human Herpes Simplex Virus Type 1 in Confiscated Gorilla

**DOI:** 10.3201/eid2011.140075

**Published:** 2014-11

**Authors:** Kirsten V.K. Gilardi, Kristie L. Oxford, David Gardner-Roberts, Jean-Felix Kinani, Lucy Spelman, Peter A. Barry, Michael R. Cranfield, Linda J. Lowenstine

**Affiliations:** University of California, Davis, California, USA (K.V.K. Gilardi, K.L. Oxford, P.A. Barry, M.R. Cranfield, L.J. Lowenstine);; Mountain Gorilla Veterinary Project, Inc., Davis (D. Gardner-Roberts, J.-F, Kinani, L. Spelman, M.R. Cranfield)

**Keywords:** gorilla, herpes simplex virus, zoonoses, nonhuman primate, great ape, herpesvirus, Gorilla beringei,viruses

## Abstract

In 2007, we detected human herpes simplex virus type 1, which caused stomatitis, in a juvenile confiscated eastern lowland gorilla (*Gorilla beringei graueri*) that had a high degree of direct contact with human caretakers. Our findings confirm that pathogens can transfer between nonhuman primate hosts and humans.

Most emerging infectious diseases of humans are of wildlife origin (*1*). The close genetic relatedness of humans and nonhuman primates (NHPs) makes each uniquely susceptible to pathogens of the other (*2*). Spillover of NHP pathogens into humans can lead to human disease pandemics (e.g., HIV-1 from the chimpanzee strain of simian immunodeficiency virus [*3*]), and some NHP viruses are potentially acutely lethal to humans (e.g., herpes B virus of Asian macaques [*4*]). The opposite transmission event—human to NHP—is less frequently reported. Although of concern from a wildlife conservation standpoint (*5*), human-to-NHP transmission events substantiate the fact that pathogen sharing is bidirectional (*6*). To provide further evidence for the potential for disease emergence at the human–NHP interface, we report a human herpes simplex virus type 1 (HSV-1) infection in an eastern lowland gorilla (Grauer’s gorilla, *Gorilla beringei graueri*).

## The Study

**Figure 1:**
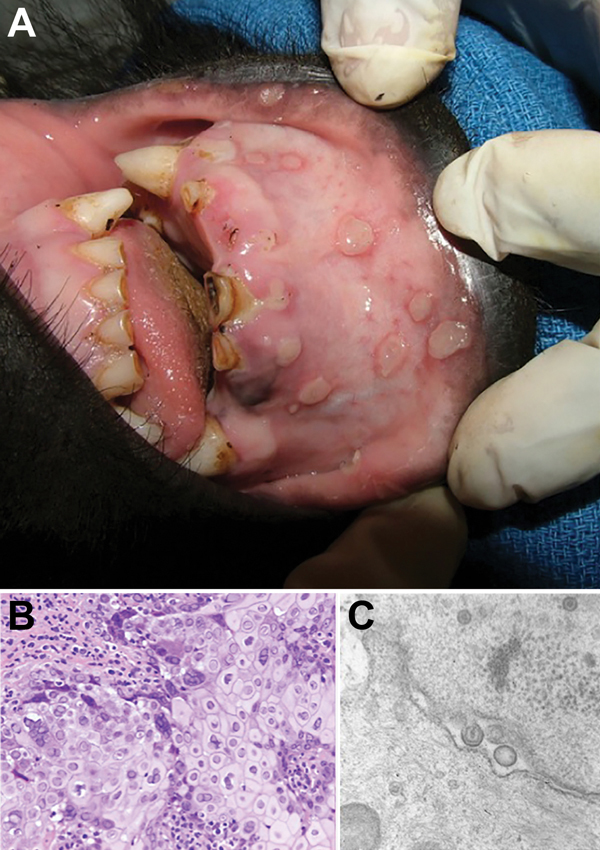
Vesicular stomatitis in a wild-caught juvenile Grauer’s gorilla (*Gorilla beringei graueri*). Gross lesions, histopathologic examination, transmission electron microscopy, and molecular screening confirmed human herpesvirus type 1 (HSV-1) as the etiologic agent. A) Human HSV-1 lip lesions in a wild-caught juvenile Grauer’s gorilla. B) Section of oral mucosa adjacent to a vesiculo-ulcerative lesion exhibits epithelial cell necrosis, cytoplasmic swelling, nuclear chromatin margination (sometimes with discrete Cowdry type A inclusions), and multinucleated syncytia typical of herpesviral cytopathic effects (hematoxylin and eosin stain). Original magnification ×200. C) Electron micrograph of the same lesion demonstrates intranuclear, unenveloped virions ≈100 nm in diameter that are budding through the nuclear membrane to form enveloped virions ≈140 nm in diameter; morphologic features of both are compatible with a herpesvirus. Original magnification ×60,000.

A 2-year-old female Grauer’s gorilla, confiscated from poachers in Goma, Democratic Republic of the Congo, in November 2005, was group-housed in a facility with 6 other juvenile Grauer’s gorillas and 1 juvenile mountain gorilla (*G.b. beringei*). All gorillas had daily direct contact with one another and with human caretakers who provided hands-on care. In March 2007, 5 of the Grauer’s gorillas exhibited bouts of lethargy and anorexia and oral lesions. Three of the affected gorillas, including the gorilla from Goma, were chemically immobilized for examination. All 3 gorillas exhibited multiple clear fluid-filled vesicles up to 2 cm in diameter that affected the mucosa of the lips and gingiva (Figure 1, panel A). Biopsy and swab samples were obtained from the lesions of the gorilla from Goma. The biopsy sample was fixed in 10% buffered formalin; the swab samples were stored frozen at –80°C, and these specimens were shipped to the United States for diagnostic testing.

Histologic examination of the biopsy sample showed variably thickened, ulcerated, nonkeratinizing, stratified squamous oral mucosae. Margins of ulcers showed marked epidermal swelling (intracellular edema, ballooning degeneration) and large numbers of epithelial syncytial cells, often with marginated chromatin and smudgy intranuclear inclusion bodies filling the nucleus (Figure 1, panel B.). Inclusion bodies were present in individual cells in the stratum basalis and stratum spinosum adjacent to the ulcers. Using transmission electron microscopy, we observed virions consistent with herpesvirus in shape and size within the nucleus of syncytial cells and budding through the nuclear membrane (Figure 1, panel C.). Histopathology and transmission electron microscopy findings were essentially identical to findings from human HSV-1 infections (*7*).

**Figure 2:**
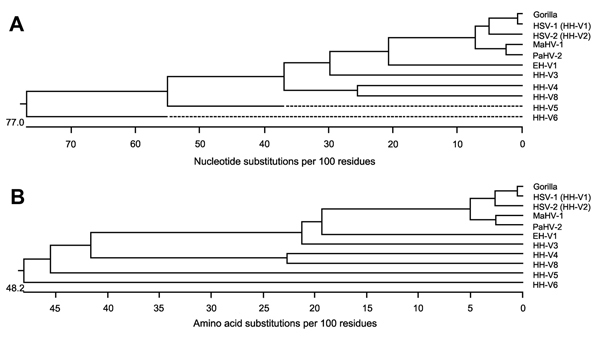
Phylogenetic analysis of the nucleotide sequence (A) and predicted amino acid sequence (B) from the swab sample amplicon from the gorilla with the corresponding regions of HSV-1 (HHV-1; GenBank accession no. AFI98948); HSV-2 (HHV-2; AGI44412); MaHV-1 (AAT67222); PaHV-2 (YP_443877); HHV-3 (varicella zoster virus; ABF21820); HHV-4 (Epstein-Barr virus; YP_401712); HHV-5 (human cytomegalovirus; AAP37469); HHV-6 (BAF93477); HHV-8 (Kaposi sarcoma virus; ACY00400), and EHV-1 ADI96155). Sequences were aligned by the Clustal W method (http://www.clustal.org) by using the MegAlign (DNAStar, Madison, WI, USA) sequence alignment program (multiple alignment parameters: gap penalty = 15.00; gap length penalty = 6.66; delay divergent seqs (%) = 30; DNA transition weight = 0.50. Pairwise alignment parameters: slow-accurate; gap penalty = 15.00; gap length = 6.66). EHV, equid herpesvirus; HSV, herpes simplex virus; HHV, human herpesvirus; MaHV, Macacine herpesvirus; PaHV, Papiine herpesvirus.

DNA was extracted from swab samples from this animal (*8*) and amplified by PCR using pan-herpesvirus–specific primers designed to the DNA polymerase gene (*UL30)* (*9*). A cloned and sequenced 731-nt amplicon (GenBank accession no. KJ396198) showed 99% identities with the DNA and protein sequences of HSV-1 *UL30*, and 94% and 95% identities (DNA and protein, respectively) to *UL30* of HSV-2 (Table). Because sequences from gorilla-specific herpesviruses have not been annotated, we performed a phylogenetic analysis of the amplicon from the gorilla swab sample using the *UL30* genes from broadly representative isolates from the 3 herpesvirus subfamilies. The gorilla-derived DNA and protein (Figure 2, panels A and B) exhibited the strongest phylogenetic relationship with HSV-1. Alignments of human and NHP alphaherpesvirus representatives show that the region amplified with the consensus herpesvirus primers is a highly conserved region of *UL30* (Technical Appendix Figure). The sequence alignments strongly suggested that the herpesvirus DNA in the oral lesion was HSV-1, although the gorilla-derived amplicon was an outlier in comparison with other HSV-1 isolates (data not shown). The relative divergence of the amplicon was due primarily to nucleotide changes encoding a 3-aa motif not found in any other annotated sequence (Technical Appendix Figure). Whether adaptation of the putative HSV-1 in the gorilla host represents a compensatory change to replication in the ectopic gorilla host is not known.

**Table:** Comparison of gorilla amplicon to other herpesviruses*

Gorilla amplicon identity	HSV-1	HSV-2	MaHV-1	PaHV-2	HHV-3	HHV-4	HHV-5	HHV-6	HHV-8	EHV-1
DNA, %	99.3	93.6	85.3	86.0	53.9	61.2	49.3	43.3	48.6	65.1
Protein, %	99.2	94.6	90.1	90.1	65.7	48.8	39.9	45.9	46.0	68.6

*Pairwise alignment of the gorilla amplicon DNA sequence (GenBank accession no. KJ396198) and predicted amino acid sequence of the corresponding region of HSV-1 (HHV-1; AFI98948), HSV-2 (HHV-2; AGI44412), MaHV-1 (AAT67222); PaHV-2 (YP_443877), HHV-3 (varicella zoster virus; ABF21820), HHV-4 (Epstein-Barr virus; YP_401712), HHV-5 (human cytomegalovirus; AAP37469), HHV6 (BAF93477), HHV-8 (Kaposi sarcoma virus; ACY00400), and EHV-1 (ADI96155). DNA–DNA alignments were performed by using the Wilbur-Lipman Method of the MegAlign (DNAStar, Madison, WI, USA) sequence alignment program (Ktuple = 3; gap penalty = 3; window = 20). Protein–protein alignments were performed by using the Lipman-Pearson method (Ktuple = 2; gap penalty = 4; gap length penalty = 12). EHV, equid herpesvirus; HSV, herpes simplex virus; HHV, human herpesvirus; MaHV, Macacine herpesvirus; PaHV, Papiine herpesvirus.

To confirm our conclusion that the lesion was associated with HSV-1 infection, we used HSV-1–specific primers to amplify a region of *UL30* (aa 134–246) outside of the amplicon defined by the pan-herpesvirus–specific primers. Amplification was positive for the oral swab sample DNA with this second primer pair and for 3 human-isolated HSV-1 strains. Sequence analysis of the amplicon demonstrated 100% and 89% sequence identity with HSV-1 and HSV-2, respectively (data not shown).

## Conclusion

On the basis of the results of the histopathologic features and molecular screening of the virus, we concluded that the stomatitis in this juvenile female Grauer’s gorilla confiscated from poachers in Goma, Democratic Republic of the Congo, was caused by HSV-1. Alphaherpesvirus exposure in wild gorillas in this region has been reported (*10*), but to our knowledge, human HSV infection in wild-born captive gorillas has not been detected with molecular techniques. Fatal HSV-1 infection in a captive-born, nursery-reared western lowland gorilla (*G. gorilla gorilla*) (*11*) has been reported. Serologic tests for HSV-1 on samples collected before the outbreak from other gorillas in the confiscated gorilla’s group showed that 2 clinically unaffected gorillas were seropositive for HSV-1, suggesting previous exposure to an alphaherpesvirus (*12*). However, serologic tests for HSV-1 and -2 exposure in the 5 clinically affected gorillas during this outbreak, including the gorilla from Goma, were negative, which suggests these animals were experiencing their first HSV infections, and antibodies were not yet detectable. Although the HSV infection status of the poachers who captured and initially held the gorilla from Goma was unknown, as was the human HSV infection status of the caretakers (from whom samples were not collected) who had daily contact with this gorilla and her captive cohort, we can safely assume most of these persons were representative of the general human population and therefore infected because HSV-1 seropositivity has been documented at a prevalence of >90% in the region (*13*–*15*), and HSV infections are lifelong.

Our findings have major ramifications for the possible reintroduction of these confiscated gorillas into the wild (all remain in captivity) because such a management action might introduce a novel (human) pathogen to a naïve population. Screening wild Grauer’s gorillas and other gorilla populations for human HSV and other human pathogens will be critical to adequately assess the risk for reintroducing captive gorillas to wild populations of these endangered great apes. From a global health standpoint, our finding of human HSV-1 in a gorilla proves once again that pathogens transfer among human and NHP hosts.

Technical Appendix Sequence alignment of the regions of *UL30* from the gorilla amplicon, herpes simplex virus (HSV) 1, HSV-2, Macacine herpesvirus 1, Papiine herpesvirus 2, and varicella-zoster virus with a majority sequence presented representing the amino acids found in most of the individual sequences.
