# Supplementary material for: Human Herpes Simplex Virus Type 1 in Confiscated Gorilla
Source: Emerg Infect Dis. 2014 Nov;20(11):1883–6. doi: 10.3201/eid2011.140075 (PMC4214296; doi:10.3201/eid2011.140075)
Supplement: Technical Appendix — Sequence alignment of the regions of UL30 from the gorilla amplicon, herpes simplex virus (HSV) 1, HSV-2, Macacine herpesvirus 1, Papiine herpesvirus 2, and varicella-zoster virus with a majority sequence presented representing the amino acids found in most of the individual sequences. [file 14-0075-Techapp-s1.pdf]

Article DOI: http://dx.doi.org/10.3201/eid2011.140075

Human Herpes Simplex Virus Type 1 in Confiscated Gorilla

Technical Appendix

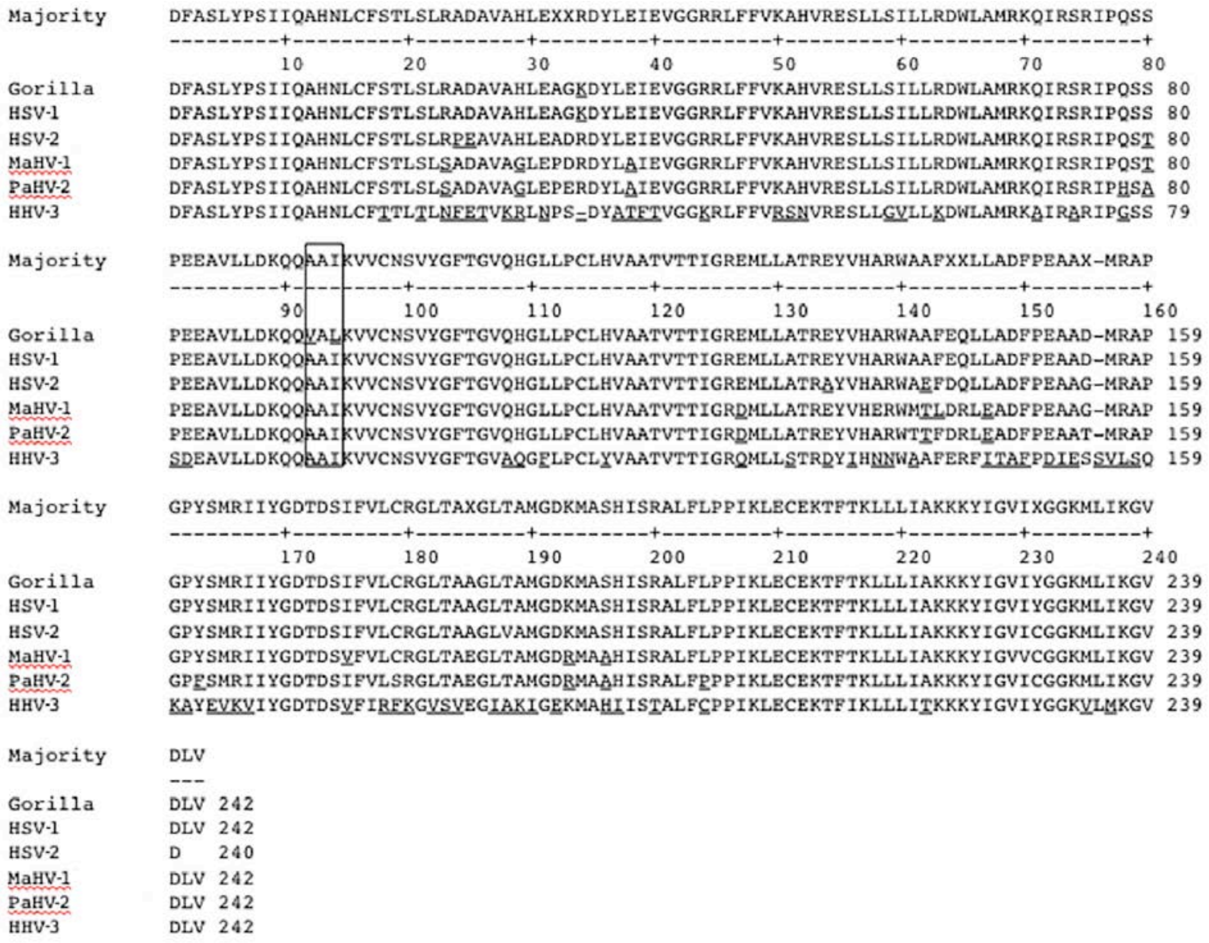

Technical Appendix Figure. Sequence alignment of the regions of *UL30* from the gorilla amplicon, HSV-1, HSV-2, MaHV-1, PaHV-2, and VZV with a majority sequence presented representing the amino acid found in most of the individual sequences. Differences from the majority sequence are underlined. The 3 aa that are boxed (aa 92–94) represent a cluster of amino acids (VAL) unique to the gorilla amplicon and not found in other alphaherpesviruses, particularly the V at position 92. However, the following 3 aa are found at the corresponding region in other HHVs: HHV-4 (LAI), HHV-5 (MAL), HHV-6 (LAL), and HHV-8 (LAI) (not shown). EHV, equid herpesvirus; HSV, herpes simplex virus; HHV, human herpesvirus; MaHV, Macacine herpesvirus; PaHV, Papiine herpesvirus.
